# Characterization of Stress Granule Protein Turnover in Neuronal Progenitor Cells Using Correlative STED and NanoSIMS Imaging

**DOI:** 10.3390/ijms24032546

**Published:** 2023-01-29

**Authors:** Stefania Rabasco, Alicia A. Lork, Emmanuel Berlin, Tho D. K. Nguyen, Carl Ernst, Nicolas Locker, Andrew G. Ewing, Nhu T. N. Phan

**Affiliations:** 1 Department of Chemistry and Molecular Biology, University of Gothenburg, SE-412 96 Gothenburg, Sweden; 2 Human Genetics, McGill University, Montreal, QC H4H1R3, Canada; 3 Faculty of Health and Medical Sciences, School of Biosciences and Medicine, University of Surrey, Guildford GU2 7XH, UK

**Keywords:** stress granules, NanoSIMS, protein turnover, neuronal progenitor cells, mass spectrometry imaging

## Abstract

Stress granules (SGs) are stress-induced biomolecular condensates which originate primarily from inactivated RNA translation machinery and translation initiation factors. SG formation is an important defensive mechanism for cell survival, while its dysfunction has been linked to neurodegenerative diseases. However, the molecular mechanisms of SG assembly and disassembly, as well as their impacts on cellular recovery, are not fully understood. More thorough investigations into the molecular dynamics of SG pathways are required to understand the pathophysiological roles of SGs in cellular systems. Here, we characterize the SG and cytoplasmic protein turnover in neuronal progenitor cells (NPCs) under stressed and non-stressed conditions using correlative STED and NanoSIMS imaging. We incubate NPCs with isotopically labelled (^15^N) leucine and stress them with the ER stressor thapsigargin (TG). A correlation of STED and NanoSIMS allows the localization of individual SGs (using STED), and their protein turnover can then be extracted based on the ^15^N/^14^N ratio (using NanoSIMS). We found that TG-induced SGs, which are highly dynamic domains, recruit their constituents predominantly from the cytoplasm. Moreover, ER stress impairs the total cellular protein turnover regimen, and this impairment is not restored after the commonly proceeded stress recovery period.

## 1. Introduction

Cellular stress can be defined as any event inflicting a strain on the homeostasis of a cell. When the cell can no longer compensate for the stress via its numerous adjustment mechanisms, the cellular stress response (CSR) is activated. The CSR aims to repair and stabilize vital macromolecules, as well as regulate energy metabolism and cell-cycle checkpoints. If the CSR fails and homeostasis cannot be restored, apoptosis will occur [1,2].

Cellular stress can occur from different sources, including chemical (e.g., toxins), physical (such as mechanical damage), or thermal (e.g., heat shock). Depending on the damage, different types of CSR exist, such as the heat shock response (HSR), the DNA damage response (DDR), and the unfolded protein response (UPR) [3,4,5]. The UPR is activated during endoplasmic reticulum (ER) stress. In ordinary physiological conditions, unfolded proteins translocate from the cytosol into the ER, where they are folded into their peculiar conformation with the assistance of chaperones. This process is highly sensitive and cellular stress can gravely impact its function. Thus, ER stress can easily lead to an accumulation of unfolded or misfolded proteins in the ER lumen. The UPR is then activated to regulate the rate of protein production and restore protein homeostasis (adaptive UPR) or trigger cell death (apoptotic UPR) [6,7,8].

Thapsigargin (TG) is a known ER stressor and UPR trigger. It acts as a sarco/endoplasmic reticulum Ca^2+^-ATPase (SERCA) pump inhibitor. The SERCA pump maintains cytosolic calcium concentration homeostasis by pumping Ca^2+^ ions into the ER lumen, and its inhibition results in the depletion of ER Ca^2+^ stores and Ca^2+^ accumulation in the cytoplasm [8,9]. This, in turn, can lead to the activation of the UPR and the formation of stress granules (SGs). SGs are a type of ribonucleoprotein (RNP) granule that are membraneless, cytoplasmic, and contain RNA–protein assemblies [10,11]. RNP granules are part of a class called biomolecular condensates, i.e., non-membrane bound condensates of different sizes which form from various assembly mechanisms via multivalent interactions. SGs comprise several macromolecular components including nucleating RNPs (e.g., RAS GTP-binding proteins, G3BP), translation initiation factors (e.g., eukaryotic translation initiation factors, eIF), ribosomal subunits, and mRNA. Their composition is also dependent on the type of stressor, but some proteins are constant such as Ras-GTPase-activating SH3-domain-binding protein 1 (G3BP1) [10]. A definite function of SGs is debated, but they are thought to protect and/or triage non-translating mRNA and proteins during cellular stress [12,13,14]. In addition to a general role during the inhibition of translation, SG composition and function can also be stress-specific [15]. Recent studies have proposed that they adopt non-homogeneous structures with variable compositions dependent on the stress and have proposed classifying SGs into three types [16]. Type I canonical SGs form via an eIF2a-dependent pathway; Type II SGs assemble following eIF2a independent inhibition of translation; and Type III SGs lack eIFs and are associated with cellular death [17]. This suggests that compositionally heterogeneous SGs support specialized functions promoting survival or pro-death outcomes. Moreover, challenging the accepted dogma that SGs consist of RNA and proteins only, we recently applied electrochemistry techniques to uncover that Type I SGs also store reactive oxygen species [18]. However, how and why different components are recruited to SGs and their impact on stress responses remain unknown.

In a healthy cell, SGs form upon stress conditions and disassemble after the stress has been resolved. The influence of chronic stress (e.g., diseases such as neurodegeneration) on the dynamics of SG aggregation and disassembly has been shown to be relevant in the recent literature. For instance, gene mutations can affect the properties of SG proteins and SG formation, causing irreversible inclusions in some disease models [19,20,21,22]. Thus, understanding SG dynamics and protein turnover can help explain some of the mechanisms by which they affect biological systems in different conditions. Protein turnover refers to the balance between protein synthesis and degradation in a biological system, in which older proteins are replaced by newly produced ones. The turnover rate of proteins in SGs and other parts of the cell under different stress and stress recovery conditions is an important parameter as it reflects not only protein metabolism, but also general cellular functions under a stress event. 

Nanoscale secondary ion mass spectrometry imaging (NanoSIMS) is an analytical technique in which a flat sample surface is eroded by a high-energy primary ion beam, generating a cloud of sputtered material including atomic and diatomic ions; these ions are then transported into a mass analyzer and a detector. Secondary ion intensity maps are then created resolving the spatial distributions of up to seven ions of interest across the sample surface. Owing to the high mass resolution and sensitivity of the NanoSIMS, isotopic ions of an element can be simultaneously detected, and their relative abundance across the sample surface can be determined [23]. To obtain the protein turnover information, cells are incubated with an isotopic amino acid (e.g., ^15^N-labelled amino acid), which is incorporated into the cells via newly synthesized proteins, followed by a sample preparation for the correlative imaging. The protein turnover of the SGs and different regions of the cells are then calculated based on the ratio of the ^15^N enrichment and the naturally abundant ^14^N content in the respective regions within the cells [24,25].

Neuronal progenitor cells (NPCs) are unipotent cells which are derived from stem cells. NPCs can differentiate within a neuronal lineage and are thus commonly used in neurodegenerative disease (ND) research. Neuronal stem cells and their derivatives appear to play an important role in strategies for disease and cellular therapy [26,27,28,29,30]. To this end, stem cell application in ND research is continuously expanding as studies investigate the multitude of cellular processes implicated in neurodegeneration and stem-cell-based therapy [28]. 

In this paper, we combined super-resolution, stimulated emission depletion (STED) microscopy with NanoSIMS imaging to characterize the protein turnover of stressed versus non-stressed wild-type (WT) NPCs derived from human-induced pluripotent stem cells (iPSCs). STED imaging was used to localize SGs in stressed cells by labelling the G3BP protein. By overlaying the STED and NanoSIMS images of the same cells, we were able to localize individual SGs in the NanoSIMS images and calculate their protein turnover. We illustrate the differences in labelled amino acid uptake and protein turnover between stressed and non-stressed cells and, by simultaneously localizing SGs and characterizing their protein turnover at the single-cell level, we identify three points where cellular stress is important. These are as follows: (1) ER stress severely affects the rate of protein turnover in the cell, i.e., cells which are stressed with TG have an impaired protein turnover regimen compared to cells that are not stressed; (2) TG-induced SGs assemble by recruiting the same ratio of existing and new proteins as the cytoplasm; and (3) TG-induced SGs keep turning over proteins during their formation and recovery at a similar rate as the rest of the cell. We demonstrate that super-resolution fluorescence microscopy and NanoSIMS imaging is a powerful integrative approach for studying the metabolic events of SGs. This provides an efficient workflow available for future investigations on the dynamics of SGs in disease models.

## 2. Results and Discussion

### 2.1. Correlative STED and NanoSIMS Imaging for Protein Turnover of SGs 

We characterized NPC protein turnover under different conditions of stress and recovery using correlative STED and NanoSIMS imaging. SGs can be identified in STED imaging via labelling against G3BP, a hallmark protein of SGs. NPCs were incubated with ^15^N-labelled leucine (hereon ^15^N leucine) which allows the tracking of protein enrichment (δ^15^N) within the cells by an increase in the ^15^N/^14^N ratio above the natural abundance ratio. The δ^15^N per mill (‰) is calculated as being relative to the atmospheric nitrogen isotopic ratio composition (r_air_ = 0.0037) using Equation (1):

(1) ∂N 15=N 15N 14rair×1000−1000

where ^15^N/^14^N is taken as ^12^C^15^N/^12^C^14^N, as the CN signal mainly originates from cell samples. ROIs were drawn on the NanoSIMS images according to the Materials and Methods, Data Analysis section. ROIs which had an enrichment with a Poisson uncertainty (as calculated in equation S1) higher than 100‰ were arbitrarily excluded for statistical value (see Figure S2). When the cells are incubated with ^15^N leucine, the amino acid is taken up and synthesized into ^15^N labelled proteins. The relative abundance of ^15^N is thus increased. 

**Figure 1:**
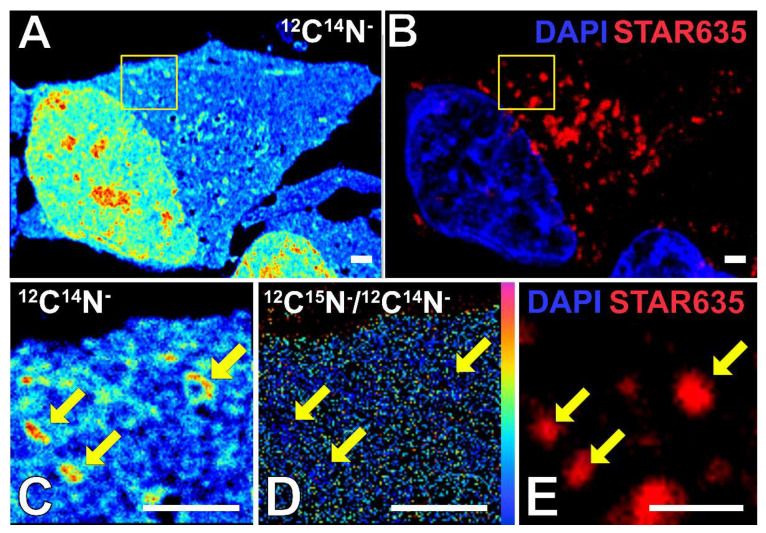
Example of correlating STED and NanoSIMS images for determining the ^15^N enrichment of SGs. (**A**) ^12^C^14^N^−^ NanoSIMS image of a stressed cell showing the shape of the whole cell and nucleus. (**B**) STED image of the stressed cell. DAPI is labelled for nucleus (blue), and anti-G3BP antibody and secondary antibody STAR 635 are labelled for SG protein G3BP (red). (**C**–**E**) Zoomed-in images of the yellow box in (**A**,**B**); (**C**) ^12^C^14^N^−^ NanoSIMS image; (**D**) ^12^C^15^N^−^/^12^C^14^N^−^ NanoSIMS image; (**E**) STED image. The arrows point to the selected SGs. Scale bars are 2 μm.

Figure 1 shows how a combination of STED and NanoSIMS imaging helps to locate SGs and determine their ^15^N enrichment. This combination of imaging approaches provides good compatibility in lateral resolution (around 50 nm) and allows precise localization of subcellular structures that cannot be identified using electron microscopy, which has been commonly used correlatively with NanoSIMS. In addition, the use of super-resolution STED enables the examining of the structure and the size of the SGs, which is useful information for the study of healthy and pathological SGs. The ^15^N enrichment of the nucleus and cytoplasm can also be obtained as shown in the figure. Each cell sample was acquired for three NanoSIMS image planes, thus ensuring the imaged thickness was within the SGs. In addition, the STED imaging performed beforehand was also set at the respective thickness as for the NanoSIMS measurements.

Control cell samples, which were not incubated with ^15^N leucine, showed no isotopic enrichment (see Figure S3, δ^15^N = −9 ± 7‰). To examine the effect of ER stress on amino acid uptake and total protein turnover, we performed experiments in which cells underwent ^15^N leucine incubation before or after stress, during stress, or before stress recovery.

### 2.2. Effect of Cellular Stress on Amino Acid Uptake and Cellular Protein Turnover

First, we looked at the effect of TG-induced ER stress on amino acid uptake and cellular protein turnover. We were interested in seeing a clear effect of ER stress on the protein turnover of the cells; thus, we used 10 μM TG, which is within the typical dose range. Cells were treated under different conditions: (i) stressed with TG for 1 h followed by 1 h incubation with ^15^N leucine (*TG → ^15^N*); (ii) concurrently stressed and incubated with ^15^N leucine for 1 h (*TG + ^15^N*); (iii) incubated with ^15^N leucine for 1 h (*^15^N*); (iv) incubated with ^15^N leucine for 1 h followed by 1 h stress (*^15^N → TG*); and (v) incubated with ^15^N leucine for 1 h followed by 1 h clearing, in which cells were incubated in regular cell media without isotopic amino acid (*^15^N → clear.*). Afterwards, the cells were chemically fixed, immunostained for the SG marker (G3BP), resin-embedded, sectioned, and imaged using STED and NanoSIMS.

**Figure 2:**
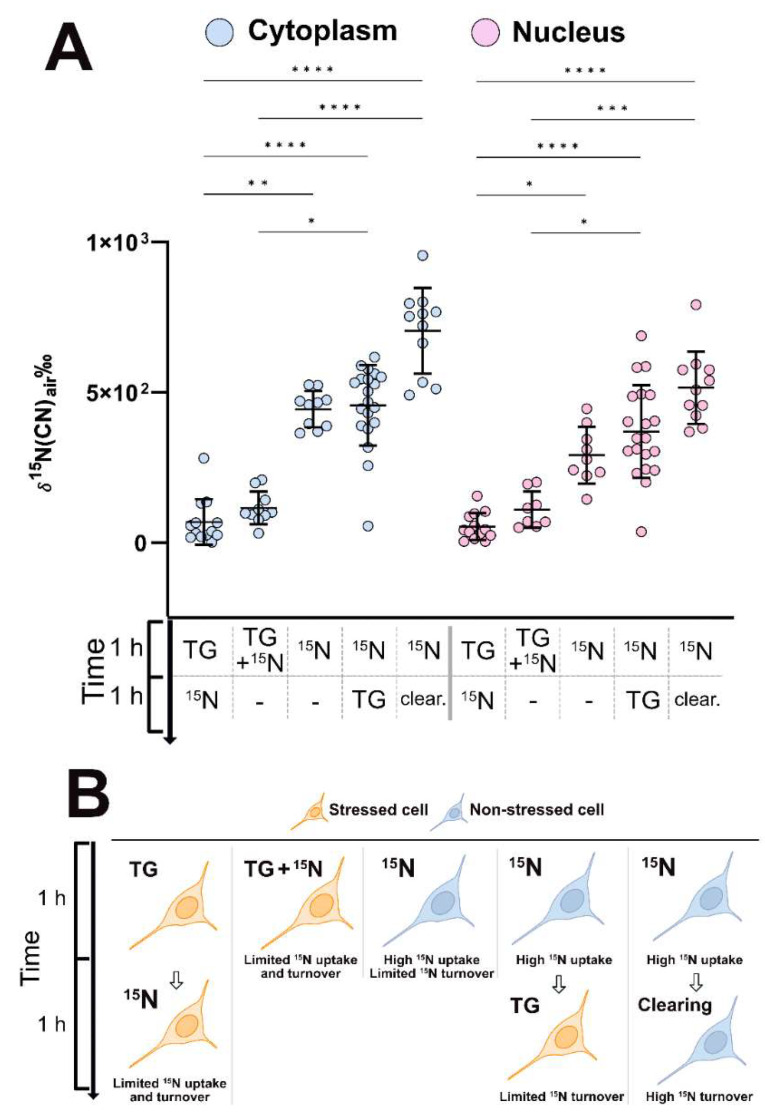
Subcellular protein turnover under the effect of ER stress. ^15^N enrichments in the cytoplasm and nucleus are significantly different between the stressed and non-stressed cells. (**A**) From left to right, for both cytoplasm (blue) and nucleus (pink): cells that had stress followed by ^15^N leucine incubation (*TG →^15^N*, n = 13), ^15^N leucine incubation and stress at the same time (*TG + ^15^N*, n = 10), only ^15^N leucine incubation (*^15^N,* n = 10), ^15^N leucine incubation followed by stress (*^15^N → TG*, n = 20), and ^15^N leucine incubation followed by clearing (*^15^N*, *clear.*, n = 11). A one-way non-parametric ANOVA finds statistical differences between the means of at least two groups in the cytoplasm and nucleus data, separately (Dunn´s, * *p* < 0.005, ** *p* < 0.01, *** *p* < 0.001, **** *p* < 0.0001); n refers to the number of cells. (**B**) Schematics of cell treatment with ^15^N incubation and TG stressor.

Significant differences in the protein turnover of the cytoplasm and the nucleus were found between different treatments (Figure 2A). The cells that were stressed before (*TG → ^15^N*) or during (*TG + ^15^N*) the ^15^N leucine incubation exhibited significantly lower turnover in both the cytoplasm and nucleus compared to those in the unstressed cells. In fact, the turnover of the stressed cells nearly decreased to the ^15^N enrichment of 0. In addition, the protein turnover of the cells being stressed after the ^15^N leucine incubation (*^15^N → TG*) was at the same level as that of the unstressed cells. This shows that the cellular protein turnover is dramatically inhibited by stress from the moment the stress occurs. Moreover, for the unstressed cells, the incubation with ^15^N leucine followed by clearing time (*^15^N → clear.*) resulted in higher turnover, albeit not statistically significantly (Dunn’s, *p* > 0.05), than the one without clearing (*^15^N*). This is likely because the cells had more time to synthesize proteins and thus a higher amount of ^15^N was incorporated into the cells during the clearing time. Again, we hypothesize that this is due to the latter having had more time to incorporate the amino acids into proteins. The mean δ^15^N of the cells which were fixed immediately after ^15^N leucine incubation (*^15^N*) is significantly higher than that of those which were stressed before being incubated with the amino acid (*TG →^15^N*); thus, the effect of fixation is not comparable to the effect of stress in terms of lowering the amount of ^15^N in the cells. Lastly, the protein turnover of the cytoplasm is higher than that of the nucleus, although not statistically significant. This can be explained by a high amount of DNA, which contains CN which is not replaced by the ^15^N leucine. However, this relation is not seen in the stressed cells at the sensitivity of the NanoSIMS measurement in a range of ppb-ppm [31], indicating the inhibitory effect of stress on the total protein turnover of the entire cells. Figure 2B graphically summarizes the sample treatments and their proposed outcomes. Cells that are firstly stressed (*TG →^15^N* and *TG + ^15^N*) have lower ^15^N enrichment, meaning protein turnover, compared to cells which are stressed after incubation or are healthy (*^15^N → TG* and *^15^N*). Healthy cells with clearing time (*^15^N → clear.*) have the highest turnover. This trend is reflected in Figure 2A and Figure S4.

### 2.3. Protein Turnover during SG Assembly

SGs are assembled to protect key cellular components during stress; however, how SGs turn over has been unclear. In this section, we examine how the turnover of SGs takes place before and during ER stress. We also look at the protein turnover of SGs compared to different cellular compartments (cytoplasm and nucleus). Cells were treated following one of the following two procedures: being incubated with ^15^N leucine for 1 h followed by 1 h of ER stress (*^15^N → TG*), or being ER-stressed for 1 h followed by ^15^N leucine incubation for 1 h (*TG → ^15^N*). The cells were chemically fixed, immunostained for the SG marker (G3BP), resin-embedded, sectioned, and imaged using STED and NanoSIMS. SG ROIs for the sample that received concurrent stress and ^15^N leucine incubation (*TG + ^15^N*) had high Poisson uncertainties (see Figure S2); thus, they were not included in this comparison.

**Figure 3:**
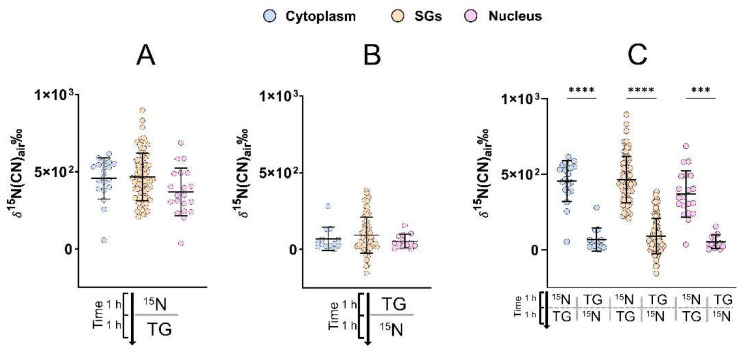
Protein turnover (expressed as ^15^N enrichment) of SGs vs. cytoplasm and nucleus in NPCs. (**A**) ^15^N enrichment in the cells that were incubated with ^15^N leucine followed by ER stress (*^15^N → TG*) (n = 20). (**B**) ^15^N enrichment in the cells that were ER-stressed followed by ^15^N leucine incubation (*TG → ^15^N*) (n = 13). (**C**) Comparison of the enrichment in the cytoplasm, SGs, and nucleus of these two groups of cells. The ^15^N enrichment compared between different cellular compartments is not significantly different, while it is significantly different compared between the two cell groups (Dunn´s, *** *p* < 0.001, **** *p* < 0.0001); n is the number of cells.

It was shown that in the *^15^N → TG* cells, the protein turnover of SGs was at the same level as in the cytoplasm, which is slightly higher than that of the nucleus (Figure 3A). However, in the *TG → ^15^N* cells, the protein turnover of SGs and the two regions were at the same level which decreases to nearly 0 (Figure 3B), revealing the inhibitory effect of the stress on cellular protein turnover. SGs appear to contain the same ratio of unlabelled and labelled CN ions, suggesting that the proteins that are being recruited into the SGs come from the same pool, and that they include new proteins which were made within the 1 h window during which the cells were incubated with ^15^N leucine before the cell stress. In addition, for Figure 3A, a Dunn´s post hoc test found no significant difference between SGs, cytoplasm, and nucleus (*p* > 0.05), but a one-way non-parametric ANOVA found a weak, statistically significant difference in ^15^N enrichment (*p* =0.047) between the cell regions. This further indicates that SGs are likely to recruit proteins from the existing cytoplasmic pool. The result suggests that SGs are highly dynamic organelles with a protein turnover comparable to that of the cytoplasm.

Moreover, significant differences were found in the protein turnover in SGs, cytoplasm, and nucleus between the two cell treatments (Figure 3C). Cells which undergo ER stress do not turn over proteins in all of their cell regions. It is evident that the ER stress inhibits the protein synthesis and turnover, which has been shown in some models [32]. This is aligned with the data shown in Figure 2A, where the healthy cells without ER stress (*^15^N*) have a ^15^N enrichment that is higher than that in the stressed cells (*TG → ^15^N*).

### 2.4. SG Protein Turnover during Stress Recovery

Cells are supposed to recover after ER stress. SGs rapidly assemble to sequester the bulk content of cytoplasmic mRNAs, and dissolve within a few hours upon stress resolution to release stored mRNAs for future translation [16,33,34]. Here, we investigate the dynamics of SG and cytoplasmic protein turnover during the recovery time after ER stress. NPCs were first incubated in a cell medium containing ^15^N leucine for 24 h, and then in a regular cell medium without the isotopic amino acid for 6 h. Afterward, the cells were stressed with TG for 1 h, followed by a recovery period in which the cells were incubated in regular cell medium. The cells were then selected for chemical fixation at different recovery time-points, i.e., after 0 h (no recovery), 30 min, and 4 h. The fixed cells were labelled for the SG marker G3BP, and further prepared for STED and NanoSIMS imaging. Because of the longer incubation time with ^15^N leucine, the ^15^N enrichment is higher (mean δ^15^N for all time-points = 8589 ± 1031‰) than the samples which were incubated in ^15^N leucine for 1 h (*^15^N → TG*, mean δ^15^N = 457 ± 134‰).

**Figure 4:**
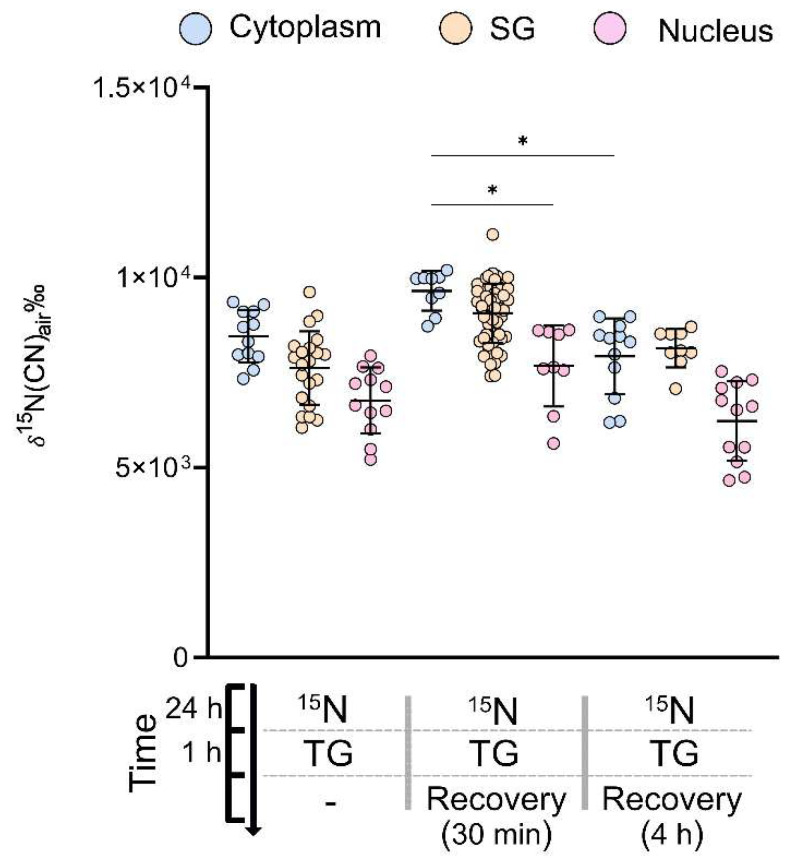
Protein turnover (expressed as ^15^N enrichment) of SGs and the cytoplasm during the stress recovery. The timeline (bottom) shows the treatment procedures for three cell groups: no recovery (n = 12), 30 min recovery (n = 9), and 4 h recovery (n = 12). A one-way non-parametric ANOVA finds statistical differences between the means of at least two groups (Dunn’s, * *p* < 0.05). Only statistical differences between the same organelles at different time-points, or different organelles at the same time-point, are shown. The mean δ^15^N for all time-points is 8589 ± 1031‰ in the cytoplasm and 8628 ± 1016‰ in the SGs; n is the number of cells.

A non-parametric ANOVA and post hoc test show no statistically significant difference between the cytoplasm and SGs in all of the samples. We deduce that the SGs are assembled by the cell using, at least in part, newly synthesized ^15^N labelled proteins. We further observed that, 30 min after stress, the number of SGs per cell was the highest (each point representing one SG in Figure 4) followed by no recovery and 4 h recovery. 

Although not statistically significant, the proteins that make up the SGs have a slightly lower amount of new amino acids than the rest of the cytoplasm, where new proteins are intended as ^15^N labelled ones that were synthesized after the isotope incubation was initiated. In addition, we note an inverse trend between the phosphorous content and the protein turnover within the cellular compartments (see Figure S5). Phosphorus is abundant in nucleic acids in DNA and RNA [35]. We speculate that a correlation might emerge between δ^15^N and phosphorous content in the long incubation samples with ^15^N leucine, as the phosphorous-rich, CN-containing materials concentrated in the SGs (e.g., mRNA) and nucleus (DNA and RNA) might contribute to the difference in the ^15^N/^14^N ratio between the cytoplasm and these compartments. This supposition needs further investigation. Additionally, the fact that they have slightly lower δ^15^N might indicate that they actively recruited some unlabelled amino acids which were taken up during the 6 h clearing time, and/or long-lived proteins which were synthesized before the isotopic incubation. To note, we do not see a significant change in protein turnover between no recovery and 4 h, and a weakly significant difference between 30 min and 4 h (Dunn´s, *p* < 0.05). This could indicate that the cell homeostasis is disrupted to the extent that the protein turnover of the cells is not yet fully recovered, even 4 h after the time the stressor is removed and the amount of SGs has decreased substantially.

## 3. Materials and Methods

*Cell Culture.* Cortical neural progenitor cells (NPCs) derived from human-induced pluripotent stem cells (iPSCs) were obtained from the Carl Ernst lab (McGill University, Montreal, Canada). The use of these human cells was approved by the Research Ethics Board of the Centre intégré universitaire de santé et de services sociaux de l’Ouest-de-l’Île-de-Montréal with the ethics approval code F9H-749. NPCs were cultured in STEMdiff neural progenitor medium (Catalog #05833, STEMCELL technologies, Vancouver, BC, Canada) on poly-D-lysine (Sigma-Aldrich, Stockholm, Sweden), laminin (Catalog #L2020, Sigma-Aldrich, Sweden) and coated T-25 flasks (Nunc™ EasYFlask™, Fisher Scientific, Lund, Sweden). Medium was exchanged every two days and cells were kept in an incubator at 37 °C and 5% CO_2_. For protein turnover experiments, NPCs were plated on Poly-D-Lysine and laminin-coated glass-bottom dishes (MatTek Life Sciences, Ashland, MA, USA). The day after plating, cells were incubated with 2 mM ^15^N-labelled leucine (Catalog P7280, Sigma Aldrich, Sweden) for either 1 h or 24 h. Stress was applied by incubating the cells with 10 μM thapsigargin (Invitrogen, Fisher Scientific, Sweden) for 1 h. G3BP was labelled in the samples as previously described by Hu et al. [18], using anti-G3BP mouse antibodies (Catalog #611126, BD Biosciences, San Jose, CA, USA), and Abberior STAR 635 anti-mouse secondary antibodies from goat (Abberior, Germany) in a dilution of 1:100. Cell nucleus was stained with DAPI (D9542, Sigma Aldrich, USA) at a concentration of 1 µg/ml for 5 min.

*Sample preparation.* Cells were fixed with 4% PFA (Fisher Scientific, Sweden) at room temperature for 30 min, then washed 6× in phosphate-buffered saline (Sigma-Aldrich, Sweden). The cells were dehydrated with increasing ethanol dilutions (30%, 50%, 70%, 85%, 95%, and 99.7%), then embedded in LR White Embedding Medium (Ted Pella Inc., Redding, CA, USA) and heat cured for 48 h. Afterwards, the embedded cells were cut into 300-nm-thick sections using an ultramicrotome (Leica EM UC6) and placed onto Si wafers (Si-Mat, Germany). Samples were kept at 4 °C and in the dark until analysis. Prior to NanoSIMS imaging, the sample sections were coated with an ultrathin layer of gold.

*STED Imaging.* An Abberior Expert Line STED microscope (Abberior, Göttingen, Germany) was used for all microscopy imaging of the cell sections. A 100× UPLSAPO NA 1.4 oil immersion objective (Olympus, Tokyo, Japan) was used for high resolution SG imaging, and a 20× UAPON NA 0.7 water immersion objective (Olympus, Japan) was used to obtain a general map of cell localization, which will be useful for identifying the target cells in NanoSIMS. The samples were put in a MatTek P35G-1.5-14-C (MatTek, Ashland, MA, USA) glass-bottom dish and imaged in two rounds. First, cells were imaged at 100×; the imaging areas containing cells were identified using the nuclei stained with DAPI to prevent imaging bias based on the presence or absence of SGs. Focus was set either on the SG in the cell labelled with STAR635 or on the nucleus stained with DAPI depending on the presence or absence of SGs. In the second round, the cells were imaged at 20× to create the cell maps. Image acquisitions were performed using the Imspector software (version 16.3.13367-w2109, Abberior, Germany). Samples from the experiments of the 24 h ^15^N leucine incubation were imaged at 30% excitation laser for both the DAPI and the STAR 635 channel. The STAR 635 channel was also imaged with a 2% STED 775 nm intensity, creating both a confocal and a STED image. The imaging was performed in a 100 × 100 μm range with 100 nm/pixel. The 1 h ^15^N leucine incubation experiment images used 50% excitation laser intensity and 3.33% STED laser intensity with a field of view of 92.4 × 89.5 μm. Due to the purpose of STED imaging being mainly used for identification of SGs and not for quantification, the imaging quality was prioritized over imaging consistency, by which a low STED intensity was used to enhance image quality while simultaneously preventing the reflection of the STED beam onto the silica wafer and the sample. For nuclei imaging with DAPI, excitation at 405 nm was used producing an emission spectrum of 415–583 nm, while SGs labelled with STAR 635 were imaged with excitation at 640 nm and detected in the emission spectrum of 650–763 nm. 

*NanoSIMS Imaging.* The measurements were performed using a 16 keV Cs^+^ source with 0.6–1 pA primary ion beam, D1–3 (200 μm width), L1 = 0 (corresponding to a beam size of 100–120 nm), and 4 ms dwell-time. At least 3 planes were acquired for every image. The entrance slit was at 20 μm width, the aperture slit was at 200 μm width, and the energy slit was fully open. The pixel size was kept between ~60–100 nm (FoV 30–50 μm, pixel size 512 × 512). A fluence of 1 × 10^17^ Cs^+^·cm^–2^ was implanted on the sample surface prior to each measurement to reach a steady state of ionization. 

*Data Analysis.* NanoSIMS images were drift-corrected and accumulated using the OpenMIMS plugin to ImageJ. One ROI containing the whole cytoplasm and one containing the whole nucleus were hand-drawn for each cell. A custom macro (see macro code S6) was run to draw ROIs around the SGs in the STED images; this included a Gaussian filter (sigma = 2), threshold (RenyiEntropy), watershed, particle analysis (size > 200 nm, circularity > 0.3), hole filling, and creating a mask. The mask images were overlaid with the corresponding STED images and cropped to fit the NanoSIMS image pixel size; the LabelsToROIs plugin [36] to ImageJ was used to create a ROI file of the SGs from the mask, and then opened with OpenMIMS. The NanoSIMS images were overlaid with the fluorescence images using Affinity Designer. The sum of pixel intensities inside the ROIs for the ^15^N/^14^N ratio was computed on the NanoSIMS images as counts/second/pixel with dead-time correction in OpenMIMS. Statistical analysis was performed using GraphPad Prism.

## 4. Limitations and Conclusions

The limitations of the study include only examining one concentration in the typical dose range of thapsigargin (1–10 μM). It is possible that lower doses of thapsigargin might affect the recovery differently and the dose dependence of the drug should be the object of future studies aimed more at recovery. Another limitation is that stress granule composition might be different based on types of stress, and different stressors will be studied in the future.

In this study, we established an imaging workflow to study the protein turnover dynamics of SGs and the effects of stress on the subcellular protein turnover in NPCs using correlative STED and NanoSIMS imaging. By combining these techniques, we can simultaneously localize SGs and characterize their protein turnover at the single-cell level. We conclude three main points: (1) ER stress severely affects the protein turnover rate in cells, i.e., cells which are stressed with TG have an impaired protein turnover regimen in subcellular regions compared to cells that are not stressed; (2) TG-induced SGs mainly assemble by recruiting the same ratio of existing and new proteins as the cytoplasm; and (3) the restoration of cellular protein turnover could take longer than a few hours of stress recovery.

We note that NanoSIMS imaging is a time- and resource-consuming technique and thus the number of samples needs to be optimized. As such, we decided to focus on only studying SG protein turnover with one common method for inducing SGs, and not on testing other parameters such as stressors (e.g., sodium arsenite, heat shock) and stressor concentrations. Thus, the present results only apply to TG-induced stress at the selected concentration in WT NPCs. Further investigations will scope ND models, different stressors, stressor parameters, and recovery times.

The results provide a new perspective on the dynamic molecular processes of SGs and their molecular relation to the cytoplasm and nucleus. In addition, the inhibition of ER stress on the cellular protein turnover was shown to last longer than expected; the latter is based on the common observation using SG markers. This implicates possibly long-lasting disruptive effects of ER stress in the cellular protein homeostasis under the conditions used, which could be a main cause for stress-related diseases and neurodegeneration.

Finally, the analytical approach developed in this study was utilized to investigate the dynamics of protein turnover within targeted organelles and protein complexes at the subcellular level, and thus can be widely applied in cell and neurobiological research.

## Data Availability

The data presented in this study are available upon request from the corresponding author.

## Supplementary Materials

The following supporting information can be downloaded at https://www.mdpi.com/article/10.3390/ijms24032546/s1.
