# Supplementary material for: Characterization of Stress Granule Protein Turnover in Neuronal Progenitor Cells Using Correlative STED and NanoSIMS Imaging
Source: Int J Mol Sci. 2023 Jan 29;24(3):2546. doi: 10.3390/ijms24032546 (PMC9917160; doi:10.3390/ijms24032546)
Supplement: Supplementary file 1 [file ijms-24-02546-s001.zip › ijms-2147724-supplementary.pdf]

Supplementary Information

$$\text{Poisson uncertainty (‰)} = \frac{1000}{\sqrt{\text{area (pixels)} \cdot {}^{12}C^{15}N\left(\frac{\frac{\text{counts}}{\text{seconds}}}{\text{pixel}}\right) \cdot \text{dwelltime (s)} \cdot \text{number of cycles}}}$$

S1. Poisson uncertainly equation. Equation used to calculate the Poisson uncertainty in each ROI in the NanoSIMS images.

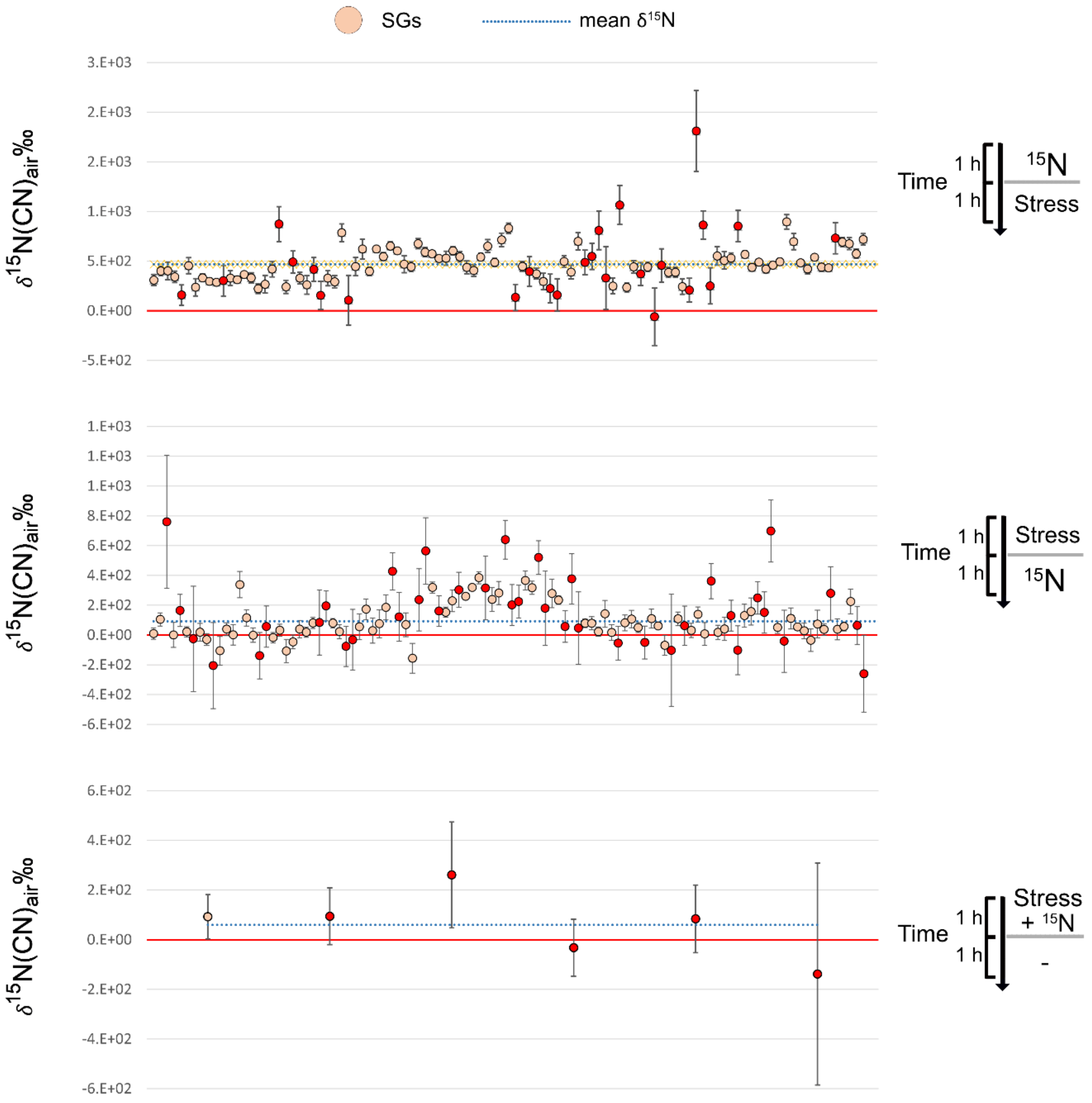

S2. ^{15}N enrichment in every SG in the stressed samples. Dots in orange represent the SGs which had Poisson uncertainties lower than 100‰ while those in red represent the SGs with Poisson uncertainties higher than 100‰ and that were thus excluded from analysis. The red line is 0‰ and blue dotted line is the mean enrichment of the SGs. The cells were stressed with thapsigargin.

S3. δ^{15}N of control cells. Control samples were not incubated with ^{15}N leucine and showed no isotopic enrichment. 5 control cells were imaged. Mean δ^{15}N is -9 ± 7‰ (standard deviation).

Cell	δ15N(CN)air‰	Poisson uncertainty ‰
1	-9	2
2	-12	3
3	-18	5
4	-1	3
5	-5	5

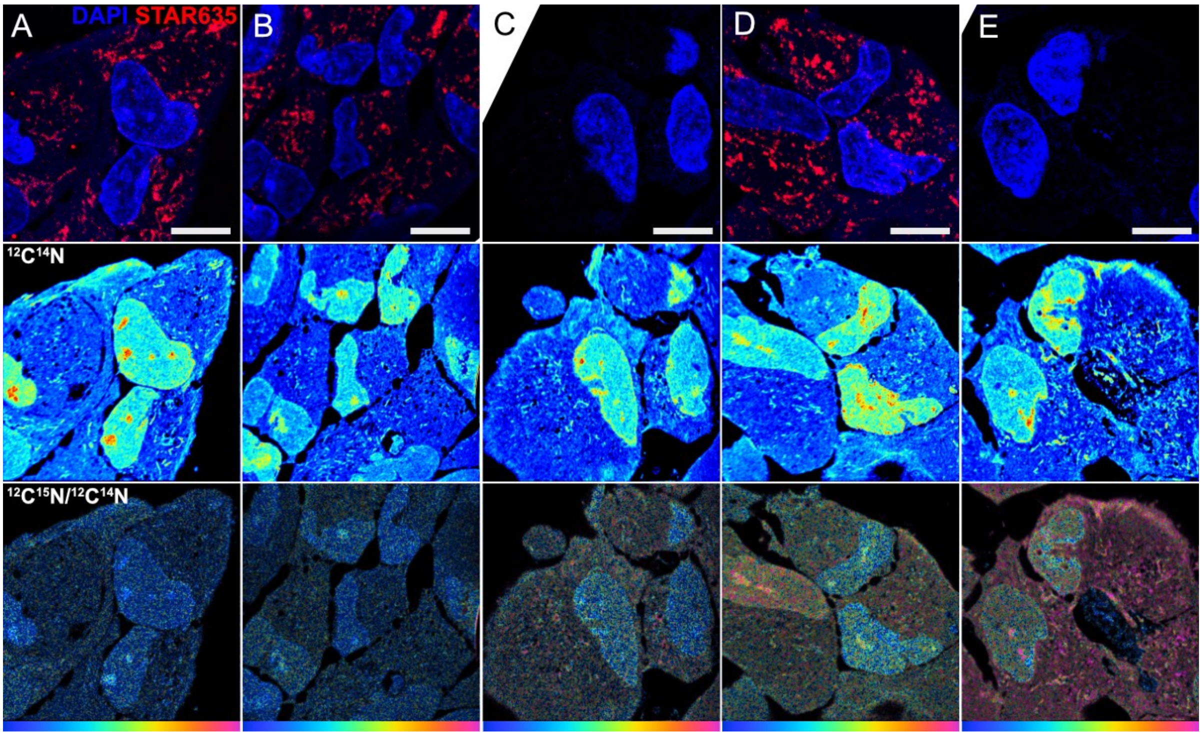

S4. Example STED and NanoSIMS correlated images of the different sample conditions. Top row: STED images, DAPI is labeled for nuclei (blue), and anti-G3BP antibody and secondary antibody STAR 635 are labeled for SG protein G3BP (red). Middle row: $^{12}C^{14}N^{-}$ NanoSIMS images showing the shape of the whole cells and nuclei. Bottom row: $^{12}C^{15}N^{-}/^{12}C^{14}N^{-}$ NanoSIMS images. Columns, A: *TG→^{15}N*. B: *TG+^{15}N*. C: *15N*. D: *^{15}N→TG*. E: *^{15}N, clear*. Scale bar is 10 µm.

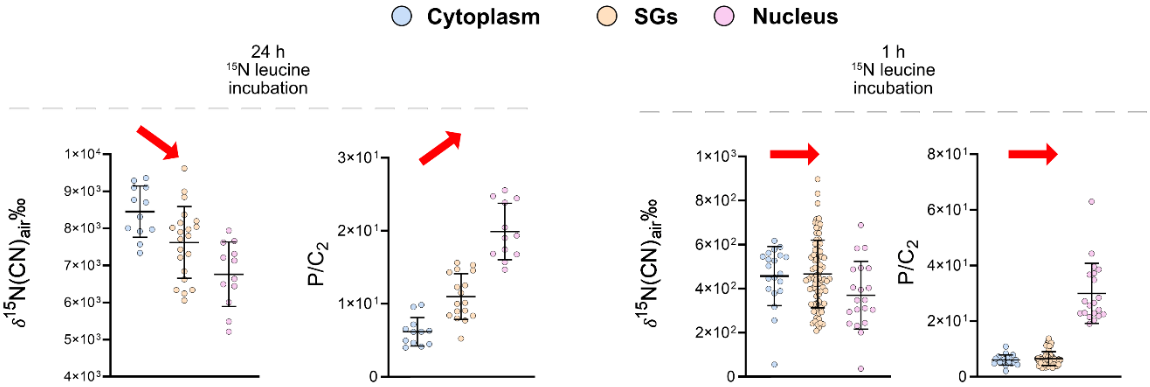

S5. $\delta^{15}N$ and P/C_2 in the cytoplasm, SGs and nucleus of the long and short ^{15}N leucine incubation samples. The SGs in the long incubation samples contain more P than the cytoplasm and this inversely reflects the $\delta^{15}N$ of the same compartments. The short incubation samples show no difference in P/C_2. This is illustrated by the red arrows. The nucleus is the compartment highest in phosphorous for both sample sets, presumably because of its high DNA content.

S6. Macros used for the data analysis. For the identification of SGs and correlation of STED images with NanoSIMS data, an ImageJ macro code was used. The code can be found at GitHub:

https://github.com/EmmBerlin/Characterization-of-Stress-Granule-Turnover-in-Correlative-STED-and-NanoSIMS/tree/main
